# A Text Book of the Diseases of Women

**Published:** 1894-09

**Authors:** 


### Reviews of Books.

*A Text Book of the Diseases of Women.* By HENRY J. GARRIGUES, M.D. Pp. 690. Philadelphia: W. B. SAUNDERS. 1894.

The first object the writer of this book has in view is to supply the wants of "the large class of physicians who have not had the advantages of a hospital training, and *who go to a post-graduate school* in order to learn gynecology"; and, secondly, he has tried to satisfy "the requirements of that much larger class who would like to go to such an establishment, but *who find it impossible to leave their practice.*" We quote from the preface because we were not aware that the gynæcological teaching amongst Americans was so unpractical, but we doubt whether this book, or any book, could possibly provide for such a lack of hospital practice.

In a treatise on Diseases of Women, we think that nearly one-fifth is an undue proportion to give to the anatomy and physiology of the female generative organs; and in the same portion of this work there appear 102 out of a total of 310 engravings and plates with which the book is usefully illustrated.

The one great fault in the work is its discursiveness. The large experience of the author is, to our mind, too little in evidence, while there is too much summarising of other people's methods, with the result that after much reading one is left in doubt as to what ought to be done in any special case. For example, in the treatment of fibroids of the uterus by supra-vaginal amputation, seven methods are given on about as many pages, but the descriptions are not supplemented by any suggestions which would make this epitome of use to one who was seeking for *practical* knowledge in the treatment of an individual case. We should like to see more of that power of guidance which comes only of experience, even though the author may have no methods of his own to tell us.

In a work which should be "up to date," we did not expect to find operation-mortality statistics (for hysterectomy, etc., p. 480) which exclude the last decade quoted as an authority for the practice of to-day; and the pathology of sarcoma and cancer, as given by the author (p. 483) is years behind the times. Pathology is often a transient dream, but when we are told that myxo-sarcoma is "also called colloid cancer," we wonder whether our dream has not become a horrible nightmare.

We are pleased to see the author is a believer in the conservative treatment of ovarian disease where such is possible. Indeed, throughout the book there is a commendable caution before recommending the resort to operation.

Three admittedly useless prescriptions are given for carcinoma uteri on p. 495, and the old error of writing "tympanitis" for "tympanites" frequently occurs.

The book shows a wide acquaintance with the subject, but the author has not done himself justice; much might be profitably removed, and its place taken by more detail and more help in discrimination, for practical uses, of that which is left.

